# Structural insights into glutathione-mediated activation of the master regulator PrfA in *Listeria monocytogenes*

**DOI:** 10.1007/s13238-017-0390-x

**Published:** 2017-03-07

**Authors:** Yong Wang, Han Feng, Yalan Zhu, Pu Gao

**Affiliations:** 1 Key Laboratory of Infection and Immunity, CAS Center for Excellence in Biomacromolecules, Institute of Biophysics, Chinese Academy of Sciences, Beijing, 100101 China; 2 University of Chinese Academy of Sciences, Beijing, 100049 China

**Dear Editor**

*Listeria monocytogenes* is a Gram-positive and facultative intracellular bacterial pathogen with two distinct lifestyles: saprophytic in the soil and parasitic in host cells (Freitag et al., 2009). *L. monocytogenes*can cause a foodborne infection characterized by bacteremia, meningoencephalitis, abortion or neonatal sepsis and a high case-fatality rate (Freitag et al., 2009). In relation to pathogenesis, the expression of most virulence genes in *L. monocytogenes* is regulated by the master regulator PrfA, which is a member of the Crp/Fnr family of site-specific DNA-binding transcription regulators (Freitag et al., 2009). The absolute requirement of PrfA for pathogenesis was demonstrated utilizing *L. monocytogenes*strains with deletions or loss-of-function mutations within the prfA gene (Chakraborty et al., 1992; Freitag et al., 1993; Leimeister-Wachter et al., 1990). PrfA activates transcription by binding to a palindromic promoter element termed the PrfA box (tTAACanntGTtAa). Very recently, glutathione (GSH), either generated by bacteria or derived from host cells, was found to be the essential small molecule cofactor of PrfA through allosteric binding to the protein (Reniere et al., 2015). PrfA^G145S^, the most well studied constitutively active mutant, was found to be able to completely bypass the requirement for glutathione during infection (Reniere et al., 2015).

Infection by *L. monocytogenes* can be detected by the cytosolic DNA sensing pathway of the host cell, thereby inducing the expression of type I interferons (IFNs) (Hansen et al., 2014). Although type I IFNs are well known for their ability to protect the host from viral infections and some bacterial infections, these pleiotropic cytokines are found to be able to exacerbate infections by *L. monocytogenes* (Rayamajhi et al., 2010). It was also found that *L. monocytogenes* is intrinsically resistant to broad spectrum cephalosporin antibiotics, which are commonly used in the treatment of bacterial infections (Krawczyk-Balska and Markiewicz, 2015). Thus a better understanding of PrfA regulation may give us an alternative strategy to control infection. Despite extensive genetic and biochemical research, the detailed molecular mechanism of PrfA activation and regulation is still unclear due to the lack of structures of PrfA bound to DNA and cofactor. In this study, we determined the crystal structures of PrfA-DNA binary and PrfA-DNA-GSH ternary complexes, thereby providing new insights into the mechanism of PrfA-mediated gene regulation.

We have co-crystallized PrfA bound to an intact 28-bp complementary dsDNA (plus 1-nt 5′ overhang at either end) and solved the structure of the binary complex at 2.93 Å resolution (X-ray statistics in Table S1). The complex contains one PrfA dimer bound to one dsDNA molecule and exhibits an intramolecular 2-fold pseudosymmetry (Fig. 1A). Each PrfA monomer consists of an N-terminal domain (aa 1–108), a long α-helical linker (aa 109–1137), and a helix-loop-helix-containing C-terminal domain (aa 138–1237) (Fig. 1A). The overall DNA bend is ~45° in the PrfA-DNA complex (Fig. 1A), which is significantly different from the previously reported bend values of approx. 80° to 90° for Crp-DNA (Benoff et al., 2002; Schultz et al., 1991) and CprK-DNA (Levy et al., 2008) complexes (Fig. S1A). Given the high quality electron density map for the DNA in the complex, we could readily build each nucleotide into the map (Fig. S1B). The intermolecular contacts between PrfA dimer and DNA (summarized in Fig. 1B) contain both specific interactions with bases and nonspecific interactions with sugar-phosphate backbone. The second helix of the helix-loop-helix motif penetrates into the major groove of the DNA, with S184 and R188 forming direct hydrogen bonds with the bases of T20 and G18, respectively (Fig. 1C). The majority of the intermolecular contacts are between PrfA and the sugar-phosphate backbone of the DNA (Fig. 1D and 1E). The superimposed structures of PrfA in DNA-bound state with either PrfA or PrfA^G145S^ in free state (Eiting et al., 2005) are shown in Fig. S1C and S1D, respectively. As expected, PrfA in the DNA-bound state is highly similar to the constitutively active mutant PrfA^G145S^ (Figs. S1D and 1G), while comparison with wild type PrfA shows significant differences in the helix-loop-helix motif and the linker region (Figs. S1C and 1F).

**Figure 1:**
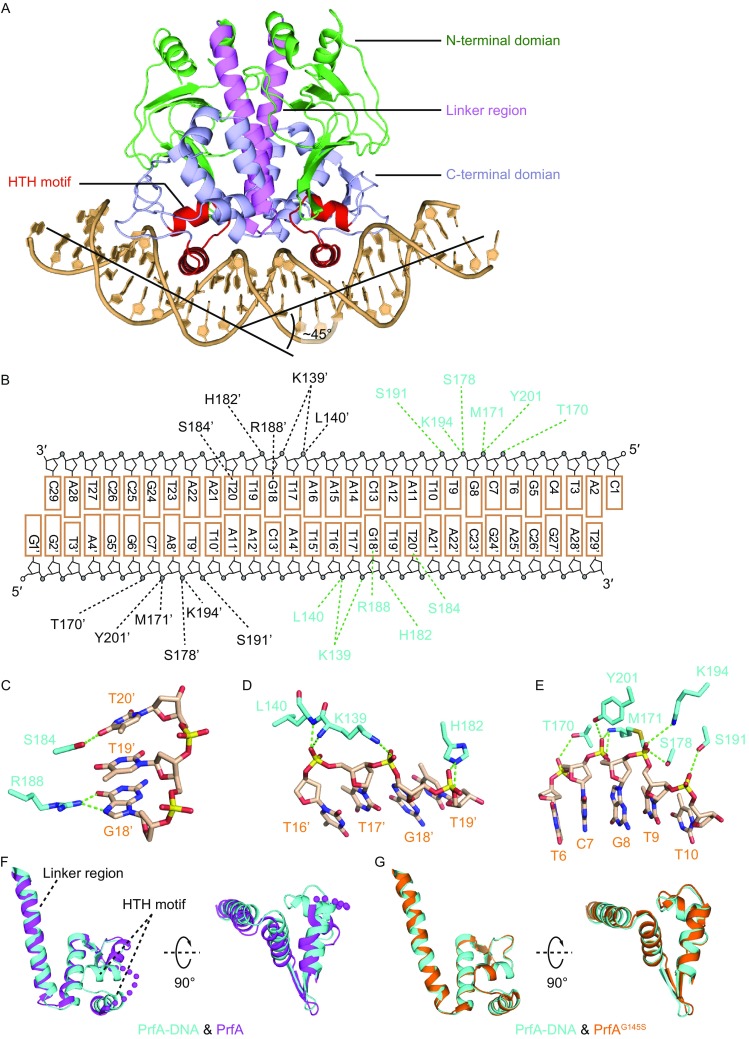
**Structure of PrfA bound to DNA**. (A) 2.93 Å crystal structure of PrfA bound to a 28 bp DNA duplex (with one base 5′ overhang at each end). N-terminal domain, C-terminal domain, and linker region are colored in green, blue and violet, respectively. The helix-loop-helix (HTH) motif in the C-terminal domain is colored in red. DNA is colored in light brown. The DNA is bent by appox. 45° upon binding to PrfA. (B) Schematic of the detailed interactions between protein and DNA. The amino acids from two PrfA monomers are colored in cyan and black, respectively. (C–E) Hydrogen bonds interactions between one PrfA monomer with the DNA. (F and G) Superposed structures of PrfA in PrfA-DNA complex (cyan) with PrfA (WT) in free state (panel F, magenta) and PrfA^G145S^ in free state (panel G, brown). The disordered region in the structure of PrfA (WT) in free state is shown as magenta dots (panel F). The two panels are shown in the same view

We have solved the structure of the PrfA-DNA-GSH ternary complex at 2.99 Å (X-ray statistics in Table S1) generated by soaking the PrfA-DNA crystal in high concentration glutathione solution. The overall structure of the ternary complex (Fig. 2A) is very similar to PrfA-DNA binary complex (Figs. 1A and S1E), with no further conformational changes detected within either protein or DNA components following glutathione binding. The glutathione molecule binds into the central cleft surrounded by N-terminal domain, C-terminal domain, and the α-helical linker (Fig. 2A). Although the detailed interactions are different, the GSH binding sites in the PrfA-DNA complex are topographically equivalent to those for cAMP in Crp (Schultz et al., 1991) (Fig. S2A), CO in CooA (Lanzilotta et al., 2000) (Fig. S2B), OCPA in CprK (Levy et al., 2008) (Fig. S2C), and 2OG in NtcA (Zhao et al., 2010) (Fig. S2D), indicating a conserved cofactor binding mode amongst the Crp/Fnr family members. The ternary complex structure also implies that the central ligand-binding cleft cannot accommodate the larger oxidized glutathione (GSSG), thereby providing a structural explanation for previous observation that PrfA does not bind to GSSG (Reniere et al., 2015). Complex formation between glutathione GSH and PrfA is mediated by van der Waals contacts and hydrogen bond interactions, whereby the glutathione molecule interacts with amino acids from both N- and C-domains, as well as the linker region (Fig. 2B and 2C). The hydrogen bonds are formed between glutathione with the main-chain of a β-strand (Y62-A66) in the N-terminal domain, the side chain of K122 and Y126 from the linker region, and side chain of Y154 from C-terminal domain (Fig. 2C). The thiol group of glutathione is embedded in a hydrophobic/aromatic pocket composed of Q61, Y63, F67, K122, Y126, and W224 (Fig. 2C).

**Figure 2:**
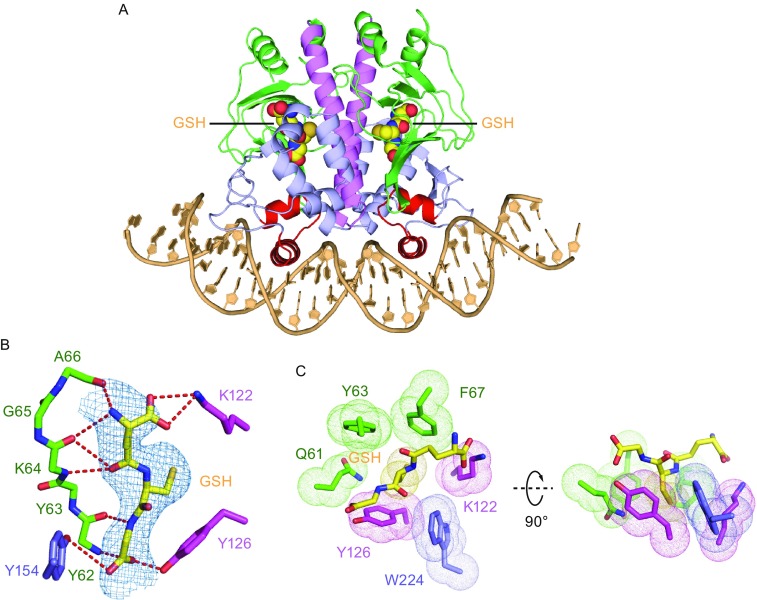
**Structure of PrfA bound to DNA and glutathione**. (A) 2.99 Å crystal structure of PrfA bound to DNA and glutathione (GSH). The color code is same as in Fig. 1A. The glutathione is shown in a space filling representation. (B) Hydrogen bonds interactions between PrfA and GSH. The color code is same as in panel A. The 2*F*o-*F*c density of GSH in yellow is shown in blue mesh with *σ* = 1.0. (C) The thiol group of GSH is embedded in a hydrophobic/aromatic pocket. The side chains of amino acids of PrfA and thiol group of GSH are shown in a dotted representation

In this study, we have provided detailed structural information on PrfA bound to its target DNA sequence and cofactor glutathione GSH. We observed a DNA-induced conformational change of PrfA by comparing the structures of PrfA in free and DNA bound states (Figs. S1C and 1F). The conformation of PrfA in the PrfA-DNA and PrfA-DNA-GSH complexes is similar to the constitutively active mutant PrfA^G145S^ in free state (Fig. S1D and 1G), consistent with the previous prediction that PrfA^G145S^ mutant adopts an induced conformation (Eiting et al., 2005). The DNA is bent by about 45° upon complex formation with PrfA, which is smaller than the previously reported approx. 80° to 90° value for Crp/Fnr family proteins: Crp and CprK (Benoff et al., 2002; Levy et al., 2008; Schultz et al., 1991). One possibility is that for PrfA-mediated gene regulation, the DNA does not need to be bent to the same degree observed for Crp and CprK complexes. Another possibility, however, is that the larger DNA bend in Crp and CprK complexes is due to the use of DNA molecules containing breaks, which may introduce artifacts during the crystallization process. In keeping with this hypothesis, an unpublished crystal structure of Crp-DNA-cAMP complex (RCSB: 3MZH) shows the same degree of bending observed for our PrfA complexes when using an intact DNA lacking breaks (Fig. S1A). Interestingly, the DNA is bent slightly larger in our structures than in the recently reported PrfA-DNA complexes (Hall et al., 2016).

It has been proposed that PrfA activation constitutes a two-step process involving initial DNA binding followed by allosteric binding of glutathione for fully transcriptional activation (Reniere et al., 2015). This implies that glutathione binding will cause additional conformational change to the PrfA-DNA binary complex. However, we did not observe conformational differences between the PrfA-DNA binary complex and the PrfA-DNA-GSH ternary complex, which is consistent with the previous understanding that PrfA could interact with DNA *in vitro* even in the absence of an activator. In addition, recent structure determination of the PrfA^G145S^-DNA complex (Hall et al., 2016) showed that there is no conformational difference between PrfA^G145S^-DNA and PrfA-DNA/PrfA-DNA-GSH complexes. Given that the constitutively active mutant PrfA^G145S^ can completely bypass the requirement for glutathione during infection (Reniere et al., 2015), we conclude that our PrfA-DNA/PrfA-DNA-GSH structures represent the fully active conformation, the same as PrfA^G145S^-DNA. More importantly, the recently solved structure of PrfA bound to GSH adopts the similar active conformation to PrfA-DNA-GSH complex, in contrast to the inactive conformation of the PrfA in free state (Hall et al., 2016). This indicates that the GSH-activated PrfA is primed for DNA binding (Hall et al., 2016). In line with the structural results, *in vitro* binding assays also confirmed that PrfA^G145S^ (structurally equivalent to PrfA-GSH) shows stronger binding to the target DNA than the wild-type PrfA (Eiting et al., 2005). Based on the structural and biochemical results, we propose that glutathione will first bind to PrfA and induce local conformational change, which is a common feature of other Crp/Fnr family members. Next, GSH-bound PrfA will bind to the target DNA to regulate gene transcription.

## Electronic supplementary material

Below is the link to the electronic supplementary material.

Supplementary material 1 (PDF 5934 kb)
