# Supplementary material for: Structural insights into glutathione-mediated activation of the master regulator PrfA in Listeria monocytogenes
Source: Protein Cell. 2017 Mar 7;8(4):308–12. doi: 10.1007/s13238-017-0390-x (PMC5359189; doi:10.1007/s13238-017-0390-x)
Supplement: Supplementary file 1 — Supplementary material 1 (PDF 5934 kb) [file 13238_2017_390_MOESM1_ESM.pdf]

SUPPLEMENTARY DATA

Protein expression and purification

The sequence corresponding to full-length PrfA were inserted into a modified pRSFDuet-1 vector (Novagen), in which PrfA was separated from the preceding His6-SUMO tag by an ubiquitin-like protease (ULP1) cleavage site. The gene sequences were subsequently confirmed by sequencing. The fusion proteins were expressed in BL21 (DE3) RIL cell strain. The cells were grown at 37°C until OD600 reached approx. 0.6. The temperature was then shifted to 20°C and the cells were induced by addition of isopropyl β-D-1-thiogalactopyranoside (IPTG) to the culture medium at a final concentration of 0.3 mM. After induction, the cells were grown overnight. The fusion protein was purified over a Ni-NTA affinity column. The His6-SUMO tag was removed by ULP1 cleavage during dialysis against buffer containing 40 mMTris-HCl, 0.3 M NaCl, 1 mM DTT, pH 7.5. After dialysis, the protein sample was further fractionated over a Heparin column, followed by gel filtration on a 16/60 G200 Superdex column. The final sample of PrfA contains about 45mg/ml protein, 20 mM Tris-HCl, 300 mM NaCl, 2 mM DTT, pH 7.5. All the mutants were cloned and purified using the same protocol as used for preparation of the wild-type protein.

Structure determination of PrfA-DNA binary complex

For crystallization of the PrfA-DNA binary complex, the sample was prepared by direct mixing protein with a 28-bp DNA (1-nt 5′-overhang at either end: upper strand 5′-GGTAGGCA**TTAACA**TT**TGTTAA**CGACGAT-3′; lower strand 5′-CATCGTCG**TTAACA**AA**TGTTAA**TGCCTAC-3′) in a 1:0.75 molar ratio. The crystals were generated by sitting drop vapor diffusion method at 20°C, from drops mixed from 0.2 μl of PrfA-DNA solution and 0.2 μl of reservoir solution (0.1 M Bis-Tris, 15% PEG3350, 0.16 M $MgCl_2$, pH 6.5). The data sets were collected at the NE-CAT beamlines of Advanced Photo Source (APS) at the Argonne National Laboratory. The diffraction data were indexed, integrated and scaled using the RAPD server of NE-CAT. The structure of PrfA-DNA binary complex was solved using the molecular replacement method in PHASER using the $PrfA^{G145S}$ structure (RCSB: 2BGC) as the search model. The model building was carried out using the program COOT and structural refinement

was carried out using the program PHENIX. The statistics of the data collection and refinement for the binary complex is shown in Table S1.

Structure determination of PrfA-DNA-GSH ternary complex

PrfA-DNA crystals for soaking in GSH solution were generated by sitting drop vapor diffusion method at 20°C, from drops mixed from 0.2 μl of PrfA-DNA solution and 0.2 μl of reservoir solution (0.1 M Tris, 27% PEG3350, 0.2 M Li_2SO_4, pH 8.5). The PrfA-DNA crystals were transferred to a fresh drop of reservoir solution with 20 mM glutathione. The crystals were picked up after soaking in the above solution for 1 h. The data sets were collected at the NE-CAT beamlines and were indexed, integrated and scaled using the RAPD server of NE-CAT. The structure of PrfA-DNA-GSH ternary complex was solved using molecular replacement method in PHASER using the PrfA-DNA binary complex as the search model. The model building was carried out using the program COOT and structural refinement was carried out using the program PHENIX. The statistics of the data collection and refinement for the ternary complex is shown in Table S1.

Table S1. X-ray Statistics of PrfA-DNA and PrfA-DNA-GSH complexes

Crystal	PrfA-DNA	PrfA-DNA-GSH
Beam line	APS-ID24C	APS-ID24C
Wavelength (Å)	0.9791	0.9791
Space group	$P2_12_12_1$	$P2_12_12_1$
Unit cell		
a, b, c (Å)	64.8, 96.4, 371.9	65.0, 96.6, 370.5
α, β, γ (^{o})	90.0, 90.0, 90.0	90.0, 99.0, 90.0
Resolution (Å)	50-2.93 (3.03-2.93)[a]	50-2.99 (3.09-2.99)[a]
R_{pim}	0.04 (0.75)	0.04 (0.96)
CC (1/2)	0.999 (0.644)	0.999 (0.703)
I/ σ (I)	13.3 (1.1)	11.4 (1.1)
Completeness (%)	99.1 (99.6)	97.8 (94.7)
Redundancy	6.3 (6.2)	6.5 (6.0)
Number of unique reflections	49838	46497
R_{work}/R_{free} (%)	21.3/26.6	23.0/28.8
R.m.s. deviations		
Bond lengths (Å)	0.008	0.011
Bond angles (^{o})	1.310	1.658

[a]Highest resolution shell (in Å) shown in parentheses.

Supplementary Figure Caption

Figure S1: Structural comparison between PrfA-DNA with other Crp/Fnr members

(A) Superposed structures of PrfA-DNA with Crp-break DNA, CrpK-break DNA, and Crp-intact DNA. Only the DNA parts are shown for clarity. DNA in PrfA-DNA complex is shown in blue, other DNAs are shown in red.

(B) The 2Fo-Fc density of DNA in the PrfA-DNA complex is shown in blue with σ=1.0.

(C, D, and E) Superposed structures of PrfA-DNA (cyan) with PrfA (WT) in free state (panel C, magenta), $PrfA^{G145S}$ in free state (panel D, brown), and PrfA-DNA-GSH (panel E, blue).

Figure S2: Ligand binding modes in other Crp/Fnr members

(A-D) The cofactor binding modes of Crp-cAMP (panel A), CooA-CO (panel B), CrpK-OCPA (panel C), and NtcA-2OG (panel D). The protein and ligands are shown in cartoon and sphere, respectively.

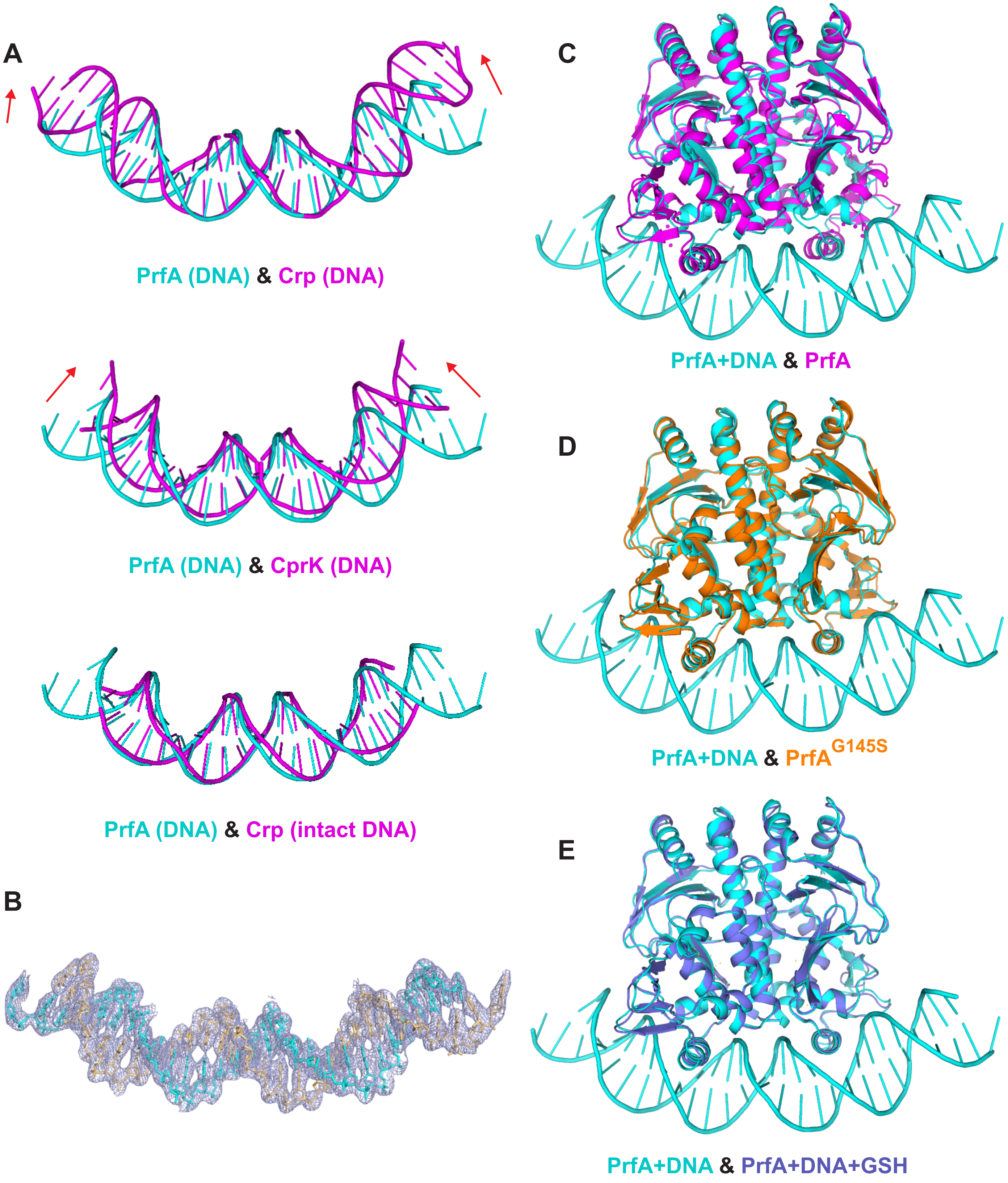
A
PrfA (DNA) & Crp (DNA)
PrfA (DNA) & CprK (DNA)
PrfA (DNA) & Crp (intact DNA)
B
C
PrfA+DNA & PrfA
D
PrfA+DNA & PrfA G145S
E
PrfA+DNA & PrfA+DNA+GSH

Figure S1

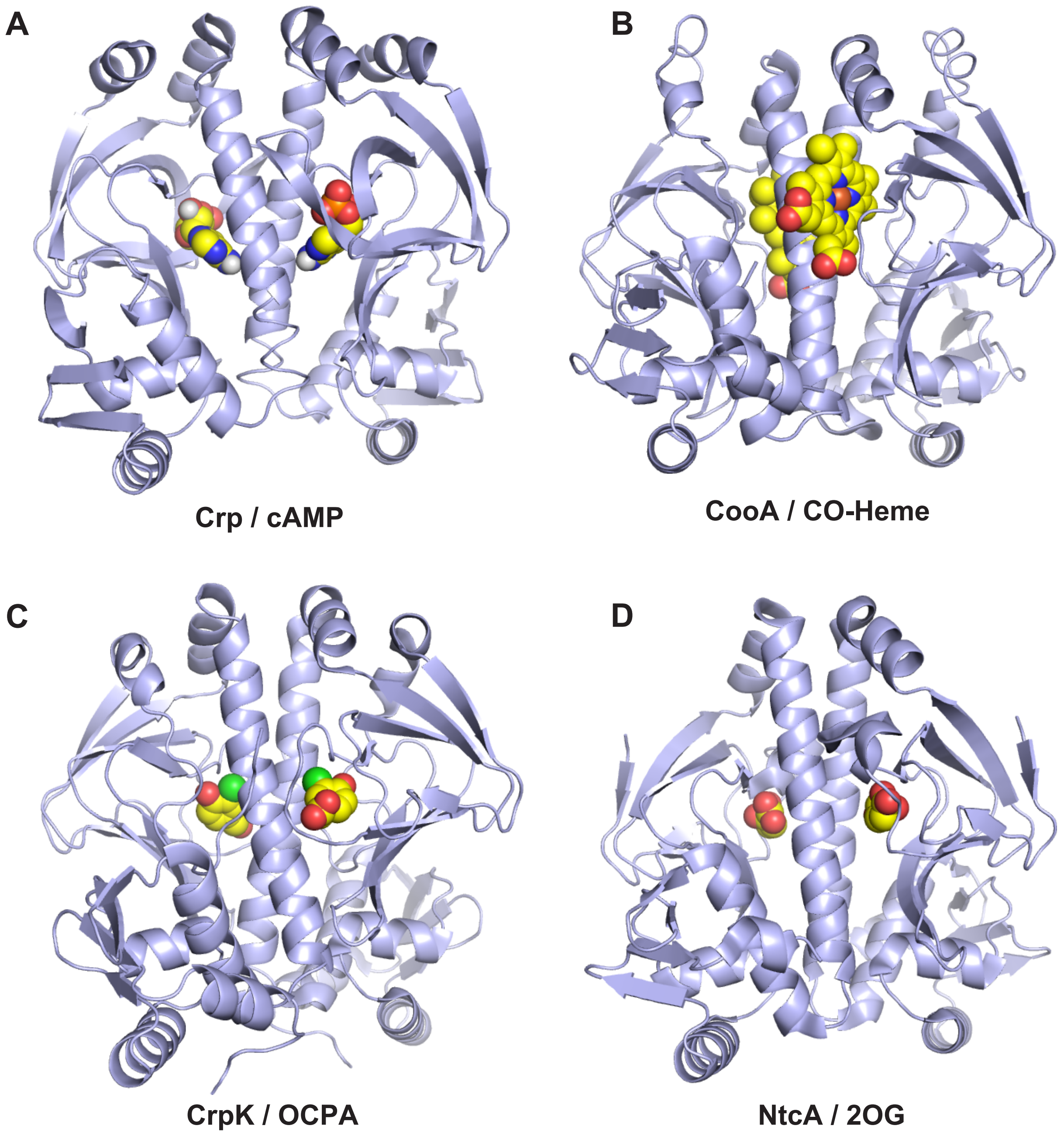

Figure S2
